# *Bowiea volubilis*: From “Climbing Onion” to Therapeutic Treasure—Exploring Human Health Applications

**DOI:** 10.3390/life13102081

**Published:** 2023-10-19

**Authors:** Hlalanathi Gwanya, Sizwe Cawe, Ifeanyi Egbichi, Nomagugu Gxaba, Afika-Amazizi Mbuyiswa, Samkele Zonyane, Babalwa Mbolekwa, Madira C. Manganyi

**Affiliations:** Department of Biological and Environmental Sciences, Botany Section, Walter Sisulu University, Nelson Mandela Drive, Mthatha Campus, Mthatha 5117, South Africa; hgwanya@wsu.ac.za (H.G.); scawe@wsu.ac.za (S.C.); iegbichi@wsu.ac.za (I.E.); ngxaba@wsu.ac.za (N.G.); ambuyiswa@wsu.ac.za (A.-A.M.); szonyane@wsu.ac.za (S.Z.); bmbolekwa@wsu.ac.za (B.M.)

**Keywords:** *Bowiea volubilis*, climbing onion, ethnobotanical uses, human health applications

## Abstract

*Bowiea volubilis* subsp. *volubilis* is primarily used to address human respiratory infections, coughs, and colds due to its diverse pharmaceutical properties. Notably, the plant contains alkaloids that exhibit notable antifungal, antibacterial, and cytotoxic properties. Additionally, the presence of saponins, with recognized antioxidant and anticancer attributes, further contributes to its medicinal potential. Steroid compounds inherent to the plant have been associated with anti-inflammatory and anticancer activities. Moreover, the bulb of *B. volubilis* has been associated as a source of various cardiac glycosides. Despite these therapeutic prospects, *B. volubilis* remains inedible due to the presence of naturally occurring toxic substances that pose risks to both animals and humans. The review focuses on a comprehensive exploration concerning *B. volubilis* ethnobotanical applications, phytochemical properties, and diverse biological activities in relation to in vitro and in vivo applications for promoting human health and disease prevention. The aim of the study is to comprehensively investigate the phytochemical composition, bioactive compounds, and potential medicinal properties of Bowiea volubilis, with the ultimate goal of uncovering its therapeutic applications for human health. This review also highlights an evident gap in research, i.e., insufficient evidence-based research on toxicity data. This void in knowledge presents a promising avenue for future investigations, opening doors to expanded inquiries into the properties and potential applications of *B. volubilis* in the context of human diseases.

## 1. Introduction

The rise in antibiotic-resistant pathogens has led to higher mortality rates for infectious diseases [1]. In the quest to explore a safer and eco-friendly therapeutic treatment for human diseases and ailments, the utilization of plant-based remedies has been well-documented in historical records through the fabrics of cultural traditions and knowledge [2]. *Bowiea volubilis* stands as a suitable candidate in the ongoing battle against antibiotic resistance and emerging infectious diseases. Although *B. volubilis* is used for various purposes, its primary application is in traditional medicine for treating respiratory infections, coughs, and colds. The plant contains various phytochemical compounds that are responsible for its medicinal properties. For example, the plant contains alkaloids, which have been reported to have antifungal, antibacterial, and cytotoxic activities [3]. Other compounds include saponins, which have been reported to have antioxidant and anticancer properties [4], and steroid compounds, including sitosterol and stigmasterol, which have been reported to have anti-inflammatory and antitumor properties [5]. In addition, flavonoid compounds such as luteolin, quercetin, and kaempferol, as well as phenolic compounds such as gallic acid, caffeic acid, and chlorogenic acid, have been reported to have antioxidant, anti-inflammatory, and anticancer properties [6].

*Bowiea volubilis* Harv. ex Hook.f. subsp. *volubilis*, commonly known as climbing onion or sea onion, is a plant species belonging to the family Asparagaceae. This plant species is native to Southern Africa, specifically South Africa, Zimbabwe, Mozambique, Botswana, and Namibia. In South Africa, *B. volubilis* grows in rocky areas, on cliffs, and on the edges of dry forests with a dry climate at elevations ranging from sea level to around 1500 m in various parts of the country, such as the Namaqualand, Drakensberg Mountains, and Gauteng Province [7,8,9,10].

This perennial herbaceous bulb-like plant consists of several distinctive parts, each with its own specific characteristics and uses [11]. The bulbous part of the stem is the most used plant part as it is traditionally used as a medicinal plant in many African cultures. The plant has long, narrow, green leaves that grow in a spiral pattern around the stem. The stem of *B. volubilis* is long, thin, and twining and is used as a support structure for the plant, allowing it to climb and reach for sunlight. The plant produces small, greenish-white flowers that are arranged in an umbel, which are not widely used but play a role in the plant’s pollination [12,13].

Environmental stressors pose a serious threat or detrimental effects on many plants [14]. *B. volubilis,* however, is an extremely adaptive plant that can tolerate a range of environmental stresses, including drought, heat, and poor soil conditions. Its ability to survive in harsh conditions is partly due to the specialized bulbous part of its stem, which stores water for extended periods. Studies have shown that the plant can mobilize various physiological mechanisms to cope with environmental stressors [15]. As such, in recent years, it has been used as a bioindicator of land restoration efforts in arid and semi-arid regions of Southern Africa [16].

While there is limited evidence-based research on its use for human consumption, there are studies suggesting the use of *B. volubilis* as a potential feed supplement for ruminants [17] due to its high content of saponins, which have been shown to have positive effects on rumen fermentation and nutrient utilization [18,19]. However, further investigation is necessary to establish the optimal dosage and potential hazards connected with administering this plant to domesticated animals.

The preparation of *B. volubilis* for treatment depends on the plant part used and the type of treatment required. These different preparation methods can ultimately affect the bioactive compounds’ content and influence the therapeutic effects of the plant. For instance, the bulb of *B. volubilis* is commonly used to treat a wide range of illnesses, including respiratory infections, gastrointestinal disorders, and skin diseases, and can be prepared in different ways, including boiling, drying, and grinding into a fine powder [20,21]. The leaves and stems of *B. volubilis* are used to treat various ailments, including hypertension, fever, and arthritis. They can be prepared by crushing or boiling, while the stems are commonly used in decoctions or infusions [22].

Even though there are numerous modern therapeutic options available worldwide, most of the global population, particularly those in rural areas, still heavily rely on herbal medicines for their health and well-being. Over 4000 plant species in South Africa, both threatened and non-threatened species, are utilized for their medicinal benefits [23]. This paper seeks to focus on the plant’s role in addressing human health issues, bridging the gap between traditional knowledge and modern scientific understanding by providing a comprehensive analysis of *B. volubilis* medicinal potential, drawing from ethnopharmacological, ethnobotanical, phytochemical, and toxicological perspectives. By achieving these aims, the research paper aims to contribute to the broader understanding of traditional herbal medicine’s relevance in modern healthcare, especially in regions where herbal remedies continue to play a vital role in supporting human health.

## 2. Methodology

**Figure 1:**
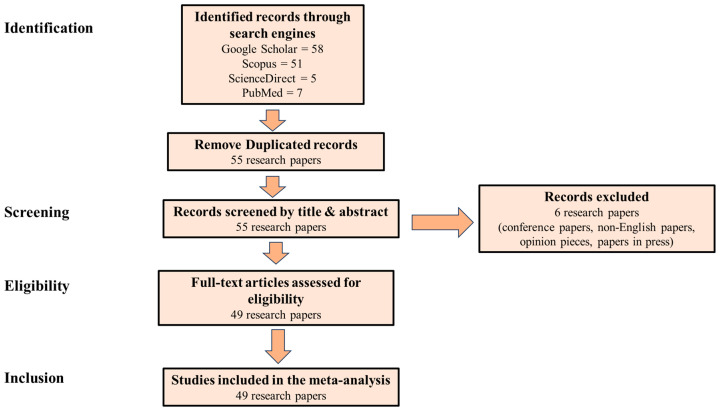
PRISMA Flowchart of studies through the systematic review process for *Bowiea volubilis* Health Applications.

High-quality global search engines such as Google Scholar, Scopus, ScienceDirect, and PubMed were used to screen, collect, review, and analyse previous research information in order to compile this current review. Keywords such as *Bowiea volubilis*, climbing onion, ethnobotanical uses and human health applications, *Bowiea volubilis*, climbing onion, and toxicity were used in the search engines to discover relevant research papers. Afterward, the abstracts were pre-screened before studying the full documents (Figure 1). Finally, the results were analysed to provide new insights into the plant’s impact on human health and its potential application in disease management.

## 3. Description, Distribution, and Habitat of *B. volubilis*

**Figure 2:**
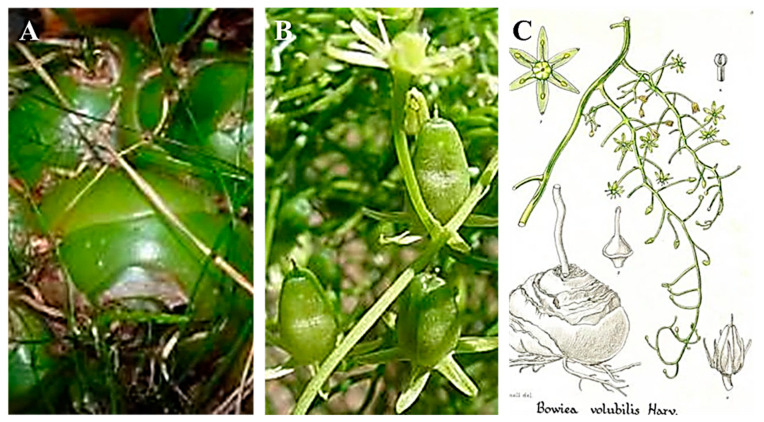
*Bowiea volubilis* (**A**) onion-like bulb, (**B**) greenish-white flowers, and (**C**) whole plant [23].

*Bowiea vobulis* is a succulent plant with a big, globose, green bulb that grows up to 15 cm in diameter, grows half buried in the ground, and sends up a twining, green-branched stem [8], as shown in Figure 2. The bulb is covered by thin branches that can wrap around anything for support [12]. It has six-petalled, half-inch wide, star-shaped, greenish-yellow flowers that develop from the top of the branches between January and February in its natural habitat and in summer in the Northern Hemisphere [13]. These green floral appendages are modified leaf petioles called cladophylls, which, like most true leaves, fall off with age and stress [8]. Since this plant does not have leaves, its photosynthesis is carried out by the stems [13]. The growth season of this plant is winter; during summer, *Bowiea volubilis* goes through a vegetative rest period [12].

*B. volubilis* is native to the grasslands and thickets of the eastern regions of South African Cape, such as the Eastern Cape, and it extends northwards all through sub-Sahara Africa (Zimbabwe, Zambia, Tanzania, and Uganda) to as far north as Kenya [8]. It has also been recorded in Mozambique, Malawi, and Angola. In South Africa, this plant is spread out in five (Eastern Cape, KwaZulu Natal, Gauteng, Mpumalanga, and North West) of the nine provinces [13]. In these regions, *B. volubilis* grows in low and medium altitudes along the mountain ranges, where it is hidden in thick river valleys, under bush clumps, and between boulder screes, where it is partially exposed to wet and dry conditions of summer [8].

## 4. Traditional Use of *B. volubilis*

The bulb of *B. volubilis* is extensively used medicinally as muthi (traditional medicine prescribed by herbalists or traditional healers (inyanga)), and native people of Southern Africa employ it for spiritual application, as they hold a profound place within the traditions of South Africa’s traditional healers [24,25]. The indigenous people value its magical abilities as they can use them to make warriors brave and unstoppable, protect travellers, and find love [15,24,26,27,28,29,30]. In addition to its magical uses, the ingestion of medicine from this bulb is also used as an antidote to poison associated with sorcery [15,29]. The stems and leaves of *B. volubilis* are commonly used for decoction due to their bright green colour and deciduous climbing nature. Several studies conducted found that numerous tribes use it as a painkiller to treat backaches, headaches, muscle pain, and pelvic pain in women [10,15,29,31,32,33,34,35,36,37,38,39]. In addition, reports indicate that *B. volubilis* is used as a blood purifier in the Limpopo and Western Cape provinces of South Africa, respectively [40,41]. The bulb also treats cancer in the Limpopo Province of South Africa [40]. Several ethnobotanical surveys have shown that the *B. volubilis* bulb relieves gastrointestinal problems [42,43,44,45]. Cimi and Campbell [46] report that the plant is used to treat kidney problems in Makhanda (former Grahamstown). The use of this plant for urinary tract infections has been reported by Philander [23], Cock [47], and Coopoosamy and Naidoo [48]. In Eswatini (former Swaziland), the bulb is cut into pieces, boiled for five minutes, and the concoction is used to treat scabies [49]. Mixing roasted bulbs with water is also used as a purgative by the Bhaca, Mfengu, and Mpondo tribes in South Africa [37,50,51]. In Transkei, a decoction of the bulb is used to treat stomach-related problems [43]. Even sexually transmitted diseases are healed using *B. volubilis* [37,50,51]. Ramarumo et al. [40] report that the plant is used as an anthelmintic. Madikizela et al. [52] list *B. volubilis* as one of the plant species used to treat tuberculosis in Pondoland, South Africa. *B. volubilis* is used as a topical medication for various skin or mucous membrane diseases [23,38,40,45,48], as well as infection of the eye [23,36,47,48]. Furthermore, liver problems are managed using *B. volubilis* [36]. Certain problems associated with pregnancy and childbirth are treated with medicines made from the bulbs of *B. volubilis* [23,35,48,53,54]. With respect to reproductive health, *B. volubilis* is used to facilitate delivery, terminate pregnancies, and treat impotence in men [30,39,44,47]. It also shows that various inflammation-associated diseases are treated with *B. volubilis* [23,34].

## 5. Phytochemistry of *B. volubilis*

**Figure 3:**
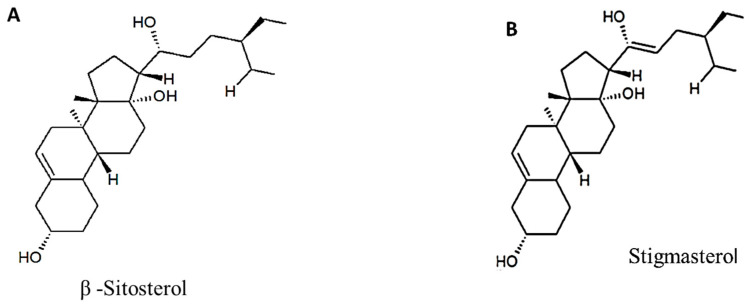

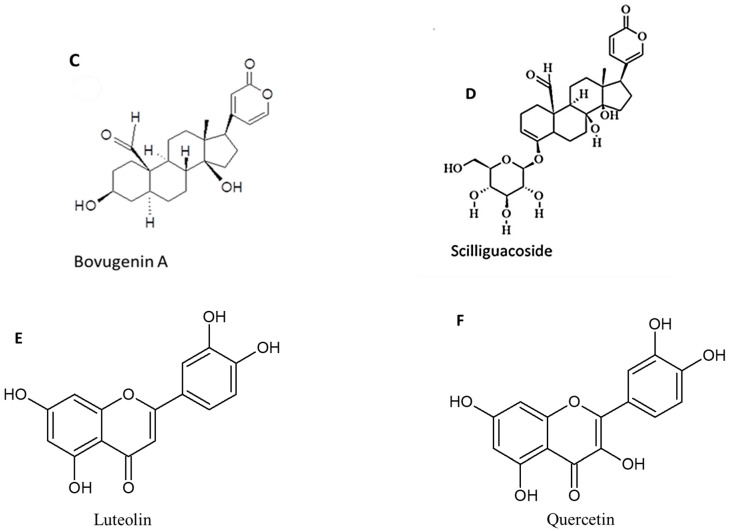
Phytochemical compounds of Bowiea volubilis, (**A**,**B**) steroids, (**C**,**D**) cardiac glycosides, and (**E**,**F**) flavonoids.

The bulb of *Bowiea volubilis* has long been known to be a source of several cardiac glycosides [42,55]. Cardiac glycosides are steroidal compounds that have proved to be fruitful in developing potential drugs for congestive heart failure [21]. Cardiac glycosides have long been isolated and characterized in *B. volubilis* [56,57,58]. These compounds consist of an aglycone or genin, which is bound to one or two sugar molecules [36,59]. Conversely, the aglycone contains an unsaturated lactone ring with either a 5-membered ring known as cardenalide or a 6-membered ring known as bufadienolide [60]. The cardiac glycoside has two classes of compounds that differ in the structure of the aglycone bovogenin A and structurally related bufadienolides [55]. A number of bufadienolides glycosides that are specific to *B. volubilis* have been isolated [61]. These include bovoruboside, sciliburoside, sciliguacoside, scillicyanoside, scilliphaeoside, bovuside A, glucobovuside, bovogenin E, and bowieasubstanz G [42]. Figure 3 presents the six phytochemical compounds of *Bowiea volubilis*. The bufadienolides present in the bulb were fractioned and characterized by LC-MS. The other bufadienolides were identified by means of thin-layer chromatography (TLC) [60], FAB-MS, NMR, and C-NMR [15].

The sugar moieties of cardiac glycosides often contain unusual 2-deoxy sugar that influences their structure, pharmacological properties, and side effects [21]. In the case of cardiac glycoside ingestion, enzymes in the body hydrolyse the glycosidic bonds, which result in the release of bioactive steroidal compounds and sugar moieties [58]. The primary pharmacological action of the cardiac glycoside is to inhibit the Na^+^/K^+^ ATPase pump and increase the intracellular Ca^2+^ levels pumped out of the cell by Na^+^/Ca^2+^ exchanger during diastole [62]. As a consequence, the intracellular Ca concentration rises, thereby inducing positive inotropy [36,42,58].

Alkaloids, which are prominently present within *B. volubilis*, have been extensively investigated for their diverse biological activities [63]. Notably, they have demonstrated efficacy in combating fungal and bacterial infections, as well as exhibiting cytotoxic effects [64]. Saponins, a class of glycosides that are known for their multifaceted health-related attributes [65], are also notably present in *B. volubilis*. These compounds, with their antioxidant and anticancer properties, contribute significantly to the plant’s therapeutic potential [66]. Furthermore, the inclusion of steroids, such as sitosterol and stigmasterol, further enhances *B. volubilis* medicinal repertoire [67]. These steroids are associated with noteworthy anti-inflammatory and antitumor capabilities, reinforcing the plant’s prospective health benefits [68]. Additionally, the presence of flavonoids, a group of phenolic compounds acclaimed for their biological significance, adds another dimension to *B. volubilis* potential therapeutic prowess. Notably, flavonoids are recognized for their antioxidant and anti-inflammatory attributes, both of which contribute to the plant’s overall health-promoting effects [69]. Within *B. volubilis*, the occurrence of specific flavonoid compounds such as luteolin, quercetin, and kaempferol further contributes to its diversified therapeutic potential [70]. Moreover, phenolic compounds, including constituents like gallic acid, caffeic acid, and chlorogenic acid, further enhance *B. volubilis* therapeutic appeal [71]. Renowned for their antioxidant, anti-inflammatory, and anticancer activities [72], these compounds provide a robust foundation for the plant’s potential role in mitigating various health-related concerns.

## 6. Biological Activity of *B. volubilis*

### 6.1. Antibacterial Activity of B. volubilis

Evidence exists on the extensive use of *B. volubilis* to traditionally treat and cure various ailments caused by pathogenic bacteria [16,50,73]. The frequent use of *B. volubilis* to treat pelvic pain, rash, liver infections, jaundice, and sexually transmitted infections has been recorded, leading one to assume that the plant has high anti-pathogenic activity [36,37,42,50]. However, studies have shown that ethanol, dichloromethane (DCM), ethyl acetate, water, and n-hexane extracts of *B. volubilis* perform poorly against bacterial pathogenic activity [16,37,50,55]. The activity of *B. volubilis* against bacteria such as *Bacillus subtilis*, *Escherichia coli*, *Klebsiella pneumoniae*, *Staphylococcus aureus*, *Oligella ureolytica, Ureaplasma urealyticum*, *Neisseria gonorrhoeae*, and *Gardnerella vaginalis*, which are implicated in the development of skin infections and on rare occasions pneumonia and meningitis as well as urogenital infections, is documented [74,75]. Masondo et al. [55] investigated the antimicrobial activity of botanically grown and muthi market-sourced *B. volubilis* against *B. subtilis*, *S. aureus*, *K. pneumoniae*, and *E. coli*. The results reported the Minimum Inhibitory Concentration (MIC) values, with the highest MIC observed in ethanol extracts against *S. aureus* as well as DCM extracts against *K. pneumoniae* and *E. coli*. In another study, Stafford et al. [16] indicated that MIC showed the highest MIC of 6.25 mg/mL against *Bacillus subtilis.* Greater activities were reported by Buwa and Van Staden [50], with a MIC value of 12.5 mg/mL for ethanol extracts in all strains, while the water solvent exhibited 3.125 mg/mL and the ethyl acetate extract showed no activity. Un-remarkable antibacterial activity of *B. volubilis* bulb and leaf tissue was also reported by Van Vuuren and co-workers [37]. Methanol, water, and DCM extracts against *Oligella ureolytica*, *Ureaplasma urealyticum*, *Neisseria gonorrhoeae*, and *Gardnerella vaginalis* showed MIC values ranging between 1.5 and 4.0 mg/mL for the CH_2_Cl_2_:MeOH (1:1 DCM and methanol) extracts and greater than 16.0 for the water extract.

### 6.2. Antifungal Activity of B. volubilis

In this current research, many studies have been reviewed on screening *B. volubilis* plants for their antifungal activity. *B. volubilis* water extracts exhibited strong inhibitory effects with a MIC value of 6.25 mg/mL against *Candida albicans* [58]. In another study, a water extract of a muthi market-sourced (MM) bulb tested against *C. albicans* showed a MIC result of 1.56 mg/mL, which was the best compared to the rest of the MIC values of other extracts [55]. Aremu et al. [42] demonstrated the activity of *B. volubilis* leaf water extracts against *C. albicans*. The results showed a distinguished MIC value of 0.5 mg/mL [42]. Discovering that bulbs can be substituted with leaves was a good indication that this plant will be available in the future. It is worth noting that, for an extract to be considered a good antifungal drug, there should be minimal drug resistance, low toxicity or minimal side effects, stability, good bioavailability, and most importantly, broad spectrum and efficacy. In addition, it has been stated that it is better for the extract to be fungicidal rather than fungistatic [9,73]. It is a well-established fact that *B. volubilis* plant extracts are significantly more effective against plant-pathogenic fungi compared to bacteria, as reported by multiple studies. Even in the early years of research, a study was conducted on 13 extracts, and only 5 extracts suppressed fungal growth, proving that, indeed, plant-pathogenic fungi are more resilient to plant extracts than plant-pathogenic bacteria [19,50,76].

### 6.3. Anti-Inflammatory Activity of B. volubilis

Several studies have proven that medicinal plants, including *B. volubilis* are an excellent source of anti-inflammatory agents [55,57]. A study conducted by Stafford et al. [16] revealed that *B. volubilis* water extracts exhibited greater performance compared to ethanol extracts when assessing the anti-inflammatory potential using cyclooxygenase (COX-1 and -2) inhibitory assays. The non-polar solvent extracts of both botanical garden-grown (BG) and muthi market-sourced (MM) *B. volubilis* bulbs showed significant inhibitory activity of greater than 70%. In the same study, it was further proven that the majority (75%) of BG extracts showed a higher percentage of inhibition compared to the MM (50%) extracts with regards to COX-1 inhibition. The inhibitory activity of the water extracts of both BG and MM bulbs against COX-2 enzymes was too small to show any activity at all, and it was found that the MM water extracts had far better COX-1 inhibitory activity compared to the BG bulbs. Results from Masondo et al. [55] showed that there was a higher COX-2 inhibition compared to COX-1 when focusing on the MM ethanol extract. An assessment of the effectiveness of various *Bowiea volubilis* bulbs extracts on the inhibition of cyclooxygenase (COX) was conducted, and it was proven that these extracts showed a high success in vitro COX assays as compared to other anti-inflammatory related enzymes, such as 5-lipoxygenase [42]. The effectiveness of *B. volubilis* was then confirmed against pain and anti-inflammatory-related illnesses as the above information was considered as further proof [44,55]. The variation of the results in which weak activity can be obtained may be due to the fact that the active compound(s) in certain extracts may be present in inadequate quantities [57,77].

### 6.4. Antiviral Activity of B. volubilis

Among various biological activities documented for *B. volubilis*, there is accumulating evidence of this plant species’ antiviral activities. In one study, the methanolic extract of *B. volubilis* bulbs was investigated for its antiviral activity against herpes simplex virus type 1 (HSV-1) [78]. The study found that the extract exhibited notable antiviral activity against HSV-1 in vitro, exhibiting an IC_50_ value of 0.34 mg/mL [78]. Another in vitro study also recorded the antiviral activity of *B. volubilis* crude extract against dengue virus type 2 (DENV-2) [79]. This study found that the extract exhibited antiviral activity at an EC_50_ value of 64.4 μg/mL against the test strain. However, the study also reported that the extract was cytotoxic at high concentrations, indicating that further research is needed to determine the safety and efficacy of the extract for use as an antiviral agent [79]. The crude extract of *B. volubilis* has also been evaluated for antiviral activity against the HIV-1 strain in another study [80]. Using a peripheral blood mononuclear cell (PBMC)-based assay, the study found that the extract exhibited significant antiviral activity against HIV-1 in vitro, reducing viral replication by up to 70% at a concentration of 50 μg/mL [80]. On the other hand, Feng et al. [81] investigated the antiviral activity of extracts from the bulb of *B. volubilis* against the respiratory syncytial virus (RSV). The study found that the extracts exhibited significant antiviral activity against RSV, with the most active extract showing an IC_50_ value of 0.13 μg/mL [81].

**Figure 4:**
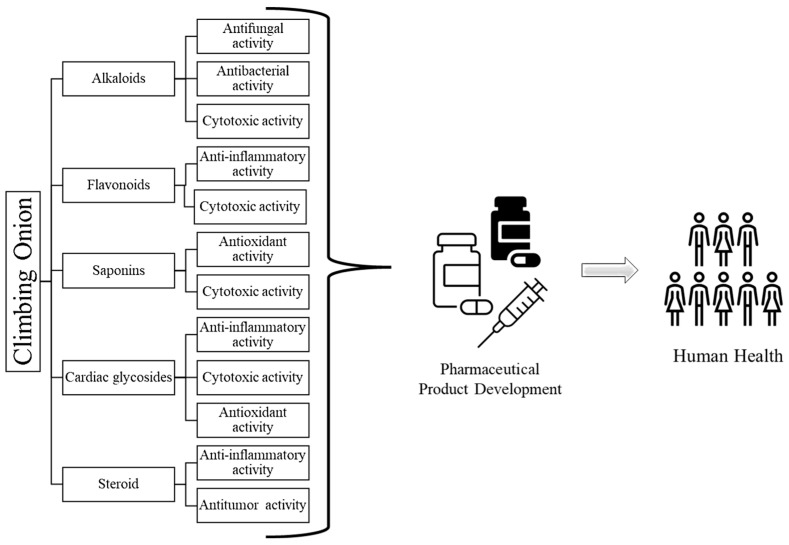
A flow chart representing various bio-compounds isolated from *Bowiea volubilis* with their activities.

While *B. volubilis* has been traditionally used in some cultures for medicinal purposes, there is insufficient scientific research specifically focused on its antiviral properties. Nevertheless, the findings documented thus far are promising and suggest that this species can be utilized, amongst other products, as an antiviral agent. Viral infection outbreaks in humans are becoming formidable pandemic threats [82], so there is an essential need for novel and natural antiviral agents. However, it is important to note that the antiviral activity of natural products is a complex and dynamic field of research, and the efficacy and safety of using *B. volubilis* or its extracts for antiviral purposes have not been fully established [78,79,80,81]. Further research, including in vitro and in vivo studies, is needed to adequately assess the antiviral properties of *B. volubilis* and its mechanism of action against specific viruses. Figure 4 and Table 1 summarizes bio-compounds that are associated with specific activities. Scientific validation of its diverse uses in traditional medicine has been demonstrated via antimicrobial, anti-inflammatory, and toxicity assays. The anti-inflammatory activity is promising; however, the available studies reveal usually low antibacterial activity, especially with bulb extracts. Bowiea volubilis includes cardiac glycosides and related chemicals, according to phytochemical screens; information on additional types of compounds is not yet available.

## 7. Toxicological Data of *B. volubilis*

The administration of traditional medicine is not well-documented, and it is roughly passed on by word of mouth from experienced healers to trainers or parents to children. This has led to the healthcare system ignoring this form of medication. *Bowiea volubilis* is rated as the top-selling traditional plant with a conservation status that is now vulnerable [55], which demonstrates the intensive use of the indigenous plant for medical relief. It is important to evaluate the safe levels of classified chemical responses to characterize exposure’s toxic effects and health. Generally, plants respond by defensive modification against herbivores and microorganisms by producing toxins, cytotoxins, and metabolic toxins that affect the central nervous system, brain, kidney, liver, heart, and lungs in humans and animals [73]. *B. volubilis* is highly poisonous and toxic to humans and animals due to the production of cardiac glycoside, a phytochemical aglycone (steroid) or polycyclic steroid compound linked to one or more sugar molecules by a glycosidic bond [42,61]. The poisonous effects of the climbing onion were reported as early as 1915, and human and animal post-mortems suggested death due to toxicity caused by cardiac glycoside compounds.

The toxicity of medicinal plants may be indirect (consumption by mistake, or incorrect selection or recommendation) or direct (misuse, overdose, incorrect preparations) [6]. Numerous studies suggest that cardiac glycoside compounds are the main contributor to toxicity at certain levels, with accumulative effects over time [55]. Ndhlala et al. [73] further emphasized that the growth stage and/or part of the plant, route and amount of administration, solubility of the compound, frequency of intoxication, and age and susceptibility of the victim all influence the severity of the toxicity. Cardenolides and bufadienolides are two compounds of cardiac glycoside listed by [61], while [42,55] argue that the cardiac glycoside compound groups are bovogenin A and bufadienolides. The varying cardiac glycosides, bovogenin A, bufadienolides, and cardenolides are associated with the toxicity effect caused by *B. volubilis* consumption. The cardiac glycoside inhibits the Na^+^/K^+^ ATPase pump, which increases intracellular Na^+^ concentration and, in turn, increases the intracellular Ca+ level, resulting in a positive inotropic effect [42,61]. The acquisition of toxicological information on *B. volubilis* is critical to our society, especially to our traditional society that depends on and trusts in medicinal plants. This study assesses the safety administration of medical plants by dosage evaluation and, most importantly, the risk assessment of the medicinal plant.

In the study conducted by Emamzadeh-Yazdi et al. [84], in vitro cytotoxicity assay (XTT colourimetric) using Vero cells (kidney epithelial cells of African monkey) and HEK 293 cells (human embryonic kidney) exposed to fresh and dry *B. volubilis* ethanol extract both showed toxicity (quite toxic). The fresh and dry ethanol extract exhibited toxicity on Vero cells with toxicity activity at IC_50_ of 50 μg/mL (quite toxic), while on HEK 293 cells, the dry ethanol extract yielded IC_50_ = 23.34 μg/mL (IC_50_ < 50 μg/mL; quite toxic) and the fresh ethanol extract exhibited IC_50_ = 28.83 μg/mL (IC_50_ < 50 μg/mL; quite toxic). Another in vitro cytotoxicity study conducted by Fasinu et al. [85] investigated the effect of *B. volubilis* aqueous extract on metabolic enzyme activity in HLM cells (Human liver microsomes). The results showed toxic activity. The in vitro cytotoxicity assay revealed that the extract on enzyme CYP1A2 exhibited toxicity activity at IC_50_ of 92.5 μg/mL (IC_50_ < 50 μg/mL quite toxic), while the extract on enzyme CYP2C9 was non-toxic, and the extract on enzyme CYP2C19 displayed toxicity activity at an IC_50_ of >1000 μg/mL (non-toxic). However, the extract on enzyme CYP3A4 revealed toxicity activity at an IC_50_ of 8.1 µg/mL of IC_50_ ≤ 20 µg/mL highly toxic). More in vivo studies are necessary to evaluate the toxicity activity of *B. volubilis*; it is still of utmost importance to generate sufficient toxicological data that will give an overview in terms of risk assessment, safety, and dosage evaluation. Studies report anecdotal reports on the toxicity of a plant, but no substantial evidence-based research is available. It is essential to acknowledge anecdotal reports but also to stress the lack of scientific evidence when discussing the toxicity of a plant like *B. volubilis*. Despite this, *B. volubilis* is ranked among the top 10 medicinal plants sold in South Africa and has displayed potential antiviral effects that might be used to treat human diseases. Natural compounds isolated from *B. volubilis* can show promising antiviral activity in laboratory studies; translating these findings into effective treatments for human diseases is a complex process. Rigorous scientific research, including preclinical and clinical trials, is necessary to establish the safety and efficacy of any potential treatments; nonetheless, it is a suitable candidate.

## 8. *Bowiea volubilis* as a Potential Therapeutic Drug: Addressing the Major Challenges toward Human Diseases

Respiratory infections encompass a range of diseases that affect the respiratory system, which includes the lungs, airways, and other related structures. These infections arise from diverse pathogens, encompassing viruses, bacteria, and fungi [86]. The manifestation of symptoms and associated effects vary depending on the precise infectious agent, with certain respiratory infections posing considerable challenges for effective treatment. Respiratory infections can lead to various complications, especially if not properly managed or treated. In the realm of medical intervention, challenges in treating respiratory ailments are notable due to multifaceted factors that encompass various dimensions. The rise of antibiotic-resistant strains of bacteria complicates the efficacy of conventional treatment strategies [87]. This phenomenon is particularly relevant in bacterial respiratory infections like pneumonia, where traditional antibiotics might exhibit diminished effectiveness. In underdeveloped countries, access to proper healthcare remains a challenge [88]. The presence of alkaloids and scillaren-type cardiac glycosides has been reported across all parts of *B. volubilis*, as indicated by research conducted by Mulholland et al. [89]. Cardiac glycosides, in particular, are known to act by selectively and effectively inhibiting Na^+^/K^+^-ATPase. The observed inhibitory effect on cytochrome P450 (CYP) enzymes *in B. volubilis* can be attributed to its alkaloid content, which serves as substrates for human CYPs. A separate study by Salminen et al. [90] highlighted the inhibitory potential of structurally similar alkaloids from plants on major human drug-metabolizing CYPs, including CYP3A4, CYP2D6, and CYP2C19.

The review conducted by Smith et al. [91] offers a comprehensive analysis of regionally relevant herbal medicine utilization, with a specific focus on remedies that have been advocated for COVID-19 treatment. Notably, within this context, *Bowiea volubilis* emerges as a viable and promising candidate. The study’s findings shed light on the potential role of *B. volubilis* within the framework of herbal medicine’s response to the ongoing COVID-19 pandemic. The identification of *B. volubilis* as a suitable candidate underscores its significance and merits further exploration in the pursuit of effective and holistic approaches to address respiratory ailments, including those associated with COVID-19. The utilization of *Bowiea volubilis* in the context of respiratory infections holds significance due to its potential therapeutic properties that have been traditionally recognized and are increasingly being explored through scientific investigation. While the scientific evidence is still evolving, the recognition of *Bowiea volubilis* as a potential therapeutic agent for respiratory infections underscores the need for continued research and exploration to establish its efficacy, safety, and potential integration into healthcare practices.

Coughs and colds are prevalent respiratory illnesses characterized by symptoms such as nasal congestion, sore throat, sneezing, and coughing. While primarily caused by viral infections, bacterial infections, and environmental factors can also contribute to their onset. These conditions often result in discomfort, impaired daily functioning, and increased healthcare utilization [92,93]. The management of coughs and colds revolves around alleviating symptoms and preventing complications. Traditional medicinal plants have been explored for their potential to provide relief from these ailments. *Bowiea volubilis*, known for its ethnobotanical uses, has drawn attention due to its bioactive compounds with potential therapeutic properties.

Gastrointestinal disorders encompass a diverse range of conditions that affect the digestive tract, including the stomach, intestines, liver, gallbladder, and pancreas. These disorders can manifest with symptoms such as abdominal pain, bloating, diarrhea, constipation, nausea, and vomiting. The causes of gastrointestinal disorders vary and can include infections, inflammation, dietary factors, genetic predisposition, and lifestyle choices [94,95]. Managing these disorders requires a multifaceted approach that addresses both symptom relief and underlying causes. Traditional medicinal plants like *Bowiea volubilis* have been explored for their potential to alleviate gastrointestinal symptoms and promote digestive health [96]. Research into the effects of *B. volubilis* on gastrointestinal disorders is still emerging. Investigating its potential impact on inflammation, gut motility, and microbial balance could provide insights into its suitability as a complementary or alternative therapeutic option [97]. While there is a potential role for *Bowiea volubilis* in gastrointestinal health, it is important to emphasize the need for comprehensive research to establish its effectiveness, safety, and appropriate usage.

Cancer is a complex and multifaceted disease characterized by uncontrolled cell growth and the potential to invade other tissues and organs [98,99]. Despite significant progress in cancer research and the development of various anti-cancer drugs, the effectiveness can be limited due to factors such as drug resistance, adverse side effects, and incomplete tumor eradication. Additionally, cancer cells can evolve by developing mechanisms to evade the effectiveness of drugs, leading to treatment resistance and disease recurrence [100]. In this context, the exploration of medicinal plants as alternative or complementary treatments for cancer has gained attention. *Bowiea volubilis*, with its historical use in traditional medicine, presents a unique opportunity for investigation. The plant’s bioactive compounds, including alkaloids, saponins, and cardiac glycosides, have shown potential in various therapeutic contexts, including anti-inflammatory, antioxidant, and anti-cancer activities [101]. Research into the potential of *B. volubilis* as an alternative or adjunctive treatment for cancer is still in its early stages. Studies on its cytotoxic effects, potential mechanisms of action, and interactions with existing anti-cancer drugs could shed light on its role in cancer management. However, it is important to note that rigorous scientific investigation, including preclinical and clinical trials, is needed to establish its safety, efficacy, and appropriate usage.

Skin conditions impact a substantial portion of the global population, ranging from 30% to 70%, making them a prevalent cause for seeking medical attention in general medical practice [102]. Over 3000 distinct skin diseases, encompassing both short-term and long-lasting forms, affect people across various age groups and social backgrounds [103]. Moreover, skin diseases can have a significant impact on quality of life due to visible symptoms, discomfort, and social stigma. Furthermore, skin disorders encompass a wide spectrum of ailments that impact the integrity, visual attributes, and operational capabilities of the skin. These afflictions can stem from genetic predispositions, environmental catalysts, immune reactions, infections, and lifestyle influences. Gaining insight into the scientific facets of skin diseases entails delving into their fundamental origins, manifestations, and intricacies [104]. In line with this, inflammation serves as a pervasive characteristic shared by numerous dermatological disorders. Conditions such as eczema, psoriasis, and acne are marked by immune reactions that precipitate inflammatory processes, giving rise to manifestations of redness, irritation, and pruritus [105]. The skin microbiome, consisting of diverse microorganisms, plays a role in skin health. The implications for wound healing and protection against potential pathogens or environmental conditions highlight the crucial role of skin homeostasis. Imbalances in the microbiome can contribute to conditions such as acne [106]. *B. volubilis* is reported to contain various bioactive compounds, including alkaloids, saponins, and flavonoids, that could contribute to its anti-inflammatory properties. This potential anti-inflammatory effect could have implications for managing various inflammatory skin conditions, as well as other disorders characterized by inflammation. The potential of *Bowiea volubilis* in combatting skin pathogens is of interest due to its reported bioactive constituents that could exhibit antimicrobial properties. These properties could make *B. volubilis* a candidate for addressing skin infections caused by various pathogens, including bacteria, fungi, and other microorganisms. However, it is important to note that scientific research on its effectiveness and safety for treating skin diseases is limited.

Sexually transmitted diseases (STDs), also referred to as sexually transmitted infections (STIs) encompass a group of infections caused by various pathogens that are typically transmitted through sexual activity. These infections result in overgrowth of opportunistic bacterial microflora causing pelvic pain, vaginal discharge, penile discharge, genital ulcers, and other symptoms and indicators of STIs, in some cases, infertility [107,108]. As of 2020, the World Health Organization (WHO) approximated a total of 374 million new infections attributed to the four most prevalent STIs: chlamydia (129 million), gonorrhea (82 million), syphilis (7.1 million), and trichomoniasis (156 million) [109]. Recent models indicate that sub-Saharan Africa and the Western/Eastern Pacific regions bear a disproportionate burden of 75% of global STI control costs [109]. Despite the fact that antimicrobial resistance is a global public health problem, front-line practitioners often underestimate the effect of antibiotic-resistant STIs [110]. Medicinal plants have garnered attention as potential sources of alternative or complementary treatments for STDs. These plants contain bioactive compounds that exhibit antimicrobial, anti-inflammatory, and immunomodulatory properties, which could contribute to their effectiveness against STD-causing pathogens [111]. The potential role of *B. volubilis* in managing STDs is an area that requires thorough scientific investigation. Traditional knowledge might suggest its historical use in addressing STDs such as *B. volubilis* as a suitable alternative [112]. Drawing from the findings, it is shown that *B. volubilis* exhibits potential antifungal, antibacterial, and antiviral properties that might be useful in the treatment of STDs as well as other health complications (Table 2).

## 9. Conservation Statues of *B. volubilis*

In South Africa, most of the medicinal plants, including *B. volubilis*, are collected from the wild, and they are decreasing at an alarming rate because of extensive exploitation [12,53]. According to herbalists, *B. vobulis* is rated one of the top six medicinal species to have become scarce because of over-exploitation. This is particularly worrying if the harvestable part of the plant is a non-renewable part, such as the bulb, rhizome, and bark. In *B. volubilis*, the most used part is the bulb [28]. Studies have shown that bulbous medicinal plants, including *B. volubilis*, are at risk of going extinct because of threats like over-exploitation, habitat destruction, human settlement, and agricultural expansion [40]. This has led to this plant being categorized as a vulnerable species in the International Union for Conservation of Nature’s (IUCN) Plant Red Data list [38]. A vulnerable species is a species whose population has declined by 30 to 50% and the cause of its decline is known [26]. It has been estimated that the population of this plant has declined by 30% in the last 30 years, and the number of individual bulbs in the muthi market has decreased tremendously [40]. Together with *Siphonochilus aethiopicus* (Schweinf.), B.L. Burtt, and *Eucomis autumnalis* (Mill.) Chitt., *Bowiea volubilis* is among the top three traded medicinal plants in South Africa assigned as being rare [53].

Various conservation strategies have been described for medicinal plants. These include in situ and ex situ conservation strategies [12]. In situ conservation is described as the conservation of the threatened species in the plant’s natural habitats, with the aim of maintaining and recovering a viable population of that species in the natural environment [38]. Ex situ, on the other hand, is concerned with the conservation of a threatened species outside the plant’s natural habitat [33]. With this strategy, the threatened species is cultivated and naturalized to ensure their continued survival and sometimes to produce large quantities of planting material for use in drug development [12]. The ex situ conservation is the one that has been proposed for *B. volubilis* [53]. To assist with the ex situ conservation of *B. volubilis*, it has been proposed that these plants be cultivated from seeds with seed coats that are acid scarified [100]. Other researchers proposed that *B volubilis* be grown from vegetative propagules using bulb scales [12]. However, it has been reported that seed and bulb scale propagation are both too slow at multiplying the needed plant material [40]. They turned to the micropropagation technique with tissue culture and found that it had saved the population of *B. volubilis* [56]. Through this technique, thousands of plantlets that can be used in the cultivation of this species have been produced [12]. Although the cultivation of medicinal plants is recognized as a conservation strategy that can provide additional or alternative stocks, concerns have been raised about the potency of their active ingredients [53]. Moreover, traditional healers believe that cultivated medicinal plants are less potent than the ones collected from the wild [40].

## 10. Conclusions

Human diseases pose a significant and ongoing concern for global public health. The diverse range of diseases that affect individuals and populations worldwide can have far-reaching implications on various aspects of society, including healthcare systems, economies, and quality of life. Medicinal plants play a significant role in the management of various human diseases due to their diverse array of bioactive compounds and therapeutic properties. These plants have been used for centuries across cultures and traditions to alleviate symptoms, promote healing, and support overall well-being. *Bowiea volubilis* holds a significant place as a recognized and widely traded medicinal plant in Southern Africa. The findings of this investigation underscore its substantial potential, encompassing antifungal, anti-inflammatory, antibacterial, and cytotoxic attributes. Consequently, the therapeutic spectrum of *B. volubilis* positions it as a promising contender for addressing conditions linked to pain, microbial infections, and inflammation-driven ailments. Notably, the scientific landscape also indicates its historical application in treating conditions such as infertility, skin disorders, cystitis, headaches, and sexually transmitted diseases. *B. volubilis* has been of interest due to its potential applications in addressing various aspects of human health and disease. Our findings outline various human diseases such as respiratory infections, cough and colds, gastrointestinal disorders, cancer, skin conditions, and sexually transmitted diseases. In regard to this, scientific research is essential to validate its effectiveness and safety in treating specific diseases. In conclusion, this study serves as a catalyst for new avenues of drug development aimed at addressing the challenges posed by human diseases and improving overall health outcomes. With the plant being threatened in the wild, conservation strategies aimed at continuously making this plant available for future use are only limited to cultivation of the plant ex situ conservation. Therefore, further research is needed to explain the specific conservation measures that can be taken to protect the *B. volubilis* population, especially in light of its potential contributions to medicine and our understanding of human diseases. The unique chemical compounds found within *B. volubilis* have demonstrated promising pharmacological properties, and their potential applications in treating or preventing various human diseases remain largely untapped.

## Figures and Tables

**Table 1:** Biological properties of *B. volubilis* with its associated bio-compounds and activity levels.

Plant Part	Extraction Solvent	Bioactive Compounds	Biological Properties	Activity Level	Ref.
**Bulb**	Water	N/A	Antifungal	>25 mg/mL	[12]
**Bulb**	Water	N/A	Antibacterial	>16.0 mg/mL	[37]
**Bulb**	Methanol	N/A	Antibacterial	1.4–4.0 mg/mL	[37]
**Bulb, leaves**		Cardiac glycosides	Anti-inflammatory		[38]
**Leaf**	Petrolium ether	Glycosides of bovogenin A	Antifungal	0.5 mg/mL	[42]
**Bulb**	Water	N/A	Antibacterial	>12.5 mg/mL	[50]
**Bulb**	Ethanol	N/A	Antibacterial	3.125 mg/mL	[50]
**Bulb**	Ethyl acetate	N/A	Antibacterial	No value	[50]
**Bulb**	Water extract	Cardiac glycoside	Antibacterial		[54]
**Bulb**	Ethanol	Cardiac glycoside	Antifungal	3.13 mg/mL (BG) and 12.50 mg/mL (MM)	[55]
**Bulb**	Ethanol	Cardiac glycoside	Antibacterial	1.56–6.25 mg/mL	[72]
**Bulb**	Petroleum ether	Cardiac glycoside	Antifungal	12. 50 mg/mL (BG and MM)	[73]
**Bulb**	Dichloromethene (DCM)	Cardiac glycoside	Antifungal	12.50 mg/mL (BG and MM)	[73]
**Bulb**	Water	Cardiac glycoside	Antifungal	3.13 mg/mL (BG) and 1.56 mg/mL (MM)	[73]
**Bulb**	Petroleum ether	Cardiotoxic glycosides of the bufadienolide group	Anti-inflammatory	COX-1 = 100% (MM)	[73]
**Bulb**	Petroleum ether	Cardiotoxic glycosides of the bufadienolide group	Anti-inflammatory	COX-2 = 100% (BG)	[73]
**Bulb**	Methanol extract	N/A	Antiviral	IC_50_ = 0.34 mg/mL	[78]
**Bulb**	Aqueous extracts	N/A	Antiviral	IC_50_ = 0.13 μg/mL	[81]
**Bulb**	Ethanol	Prostaglandin	Anti-inflammatory	COX-1 = 100%	[83]
**Bulb**	Water	Cardiac glycosides	Anti-inflammatory	COX-1 = 45%	[83]

BG: botanical garden-grown, MM: muthi market-sourced.

**Table 2:** An overall summary of the health benefits of *B. volubilis*.

Respiratory infections	Lungs, airways, and other related structures
	Pneumonia
	COVID-19
	Coughs and colds, nasal congestion, sore throat, sneezing, and coughing.
Gastrointestinal disorders	Digestive tract, including the stomach, intestines, liver, gallbladder, and pancreas Abdominal pain, bloating, diarrhoea, constipation, nausea, and vomiting
Cancer	
Skin conditions	Eczema, psoriasis, and acne
Sexually transmitted diseases (STDs)	Antifungal, antibacterial, and antiviral properties

## Data Availability

Not applicable.
